# High Adherence to the Food Pyramid’s Recommendations Avoids the Risk of Insufficient Nutrient Intake among Farmers in Peri-Urban Kenya

**DOI:** 10.3390/nu13124470

**Published:** 2021-12-14

**Authors:** Madoka Kishino, Azumi Hida, Kenta Hara, David Nguatha Mungai, Rose Okoyo Opiyo, Hirotaka Matsuda, Yuki Tada, Kazuko Ishikawa-Takata, Kenji Irie, Yasuyuki Morimoto

**Affiliations:** 1 Department of Food and Nutritional Science, Graduate School of Agriculture, Tokyo University of Agriculture, 1–1–1 Sakuragaoka, Setagaya-ku, Tokyo 156-8502, Japan; 11321001@nodai.ac.jp; 2 Research Fellow of Japan Society for the Promotion of Science, 5-3-1 Kojimachi, Chiyoda-ku, Tokyo 102-0083, Japan; 3 Department of Nutritional Science, Faculty of Applied Bioscience, Tokyo University of Agriculture, 1-1-1 Sakuragaoka, Setagaya-ku, Tokyo 156-8502, Japan; y3tada@nodai.ac.jp (Y.T.); kt207460@nodai.ac.jp (K.I.-T.); 4 Department of International Agricultural Development, Graduate School of Agriculture, Tokyo University of Agriculture, 1–1–1 Sakuragaoka, Setagaya-ku, Tokyo 156-8502, Japan; k4hara@agrinutrihealth.com; 5 Wangari Maathai Institute for Peace and Environmental Studies, University of Nairobi, Off Kapenguria Road, Nairobi P.O. Box 30197-00100, Kenya; mungaidavid@uonbi.ac.ke; 6 School of Public Health, University of Nairobi, Kenyatta National Hospital, Nairobi P.O. Box 30197-00100, Kenya; roseopiyo@uonbi.ac.ke; 7 Department of Agricultural Innovation for Sustainable Society, Tokyo University of Agriculture, 1737 Funako, Atsugi-shi, Kanagawa 243-0034, Japan; hm206784@nodai.ac.jp; 8 Department of International Agricultural Development, Faculty of International Agriculture and Food Studies, Tokyo University of Agriculture, 1-1-1 Sakuragaoka, Setagaya-ku, Tokyo 156-8502, Japan; k3irie@nodai.ac.jp; 9 Diet Diversity for Nutrition and Health Program, Alliance of Bioversity International and International Center for Tropical Agriculture—CIAT, Nairobi P.O. Box 823-00621, Kenya; y.morimoto@cgiar.org

**Keywords:** nutritional evaluation, food-based dietary guidelines, micronutrient deficiency, Kenya

## Abstract

This study aimed to investigate whether the Kenyan Food Pyramid (FP) can evaluate excess or insufficient nutrient intake. Participants were farmers (56 men and 64 women, aged 18–60 years) in Wangige Village, Kiambu County—a peri-urban area of Kenya. Cross-sectional data were collected for demographic characteristics, physical measurements, and 2-day and 24-h dietary recalls. The average adherence level to the FP (hereafter, “FP score”) was 25.0 out of 50.0, with a minimum and maximum of 14.1 and 41.5, respectively. Energy and protein % energy ratio were significantly higher (*p* for trend < 0.05) in the higher FP score group. A higher FP score was also associated with a higher energy-adjusted micronutrient intake, and it was more likely to meet nutrient requirements. However, the higher FP score group had a higher risk of excess sodium intake (*p* for trend < 0.001). The Kenyan FP could be a useful tool for avoiding the risk of insufficient nutrient intake, but not for avoiding high energy and sodium intake. It is necessary to include appropriate evaluations to limit energy, sugar, and salt. Food groups and recommendations of the FP should be optimised according to the dietary environment of the target population so as to promote their health.

## 1. Introduction

Globally, the double burden of malnutrition, characterised by the coexistence of overnutrition—such as obesity—and undernutrition—including micronutrient deficiencies, stunting, wasting, and being underweight—is of public health importance [1]. In Kenya, communicable diseases, such as acquired immunodeficiency syndrome and lower respiratory tract infections, remain the main causes of death; however, their incidence is decreasing gradually [2]. Meanwhile, deaths due to non-communicable diseases (NCDs)—such as cardiovascular disease, cancer, and diabetes—are increasing [2]. Furthermore, deaths and disabilities attributed to metabolic risk factors—such as hypertension, high body mass index (BMI), and high blood sugar—increased significantly from 2009 to 2019 [3]. According to the STEPwise SURVEY for NCD Risk Factors in 2015, 27% of Kenyan adults were overweight or obese, and 24% of them had hypertension [4]. The increase in obesity is also evident in rural areas, with obesity among rural women rising from 4.4% in 2003 to 7.0% in 2014 [5,6]. At the same time, 29% of Kenyan women of reproductive age (15–49 years) were reported to have anaemia, which increases the risk of maternal mortality and neonatal morbidity [7,8]. One of the main causes of the double burden of malnutrition is nutrition transition, along with rapid economic growth, globalisation, and urbanisation [1]. A review of nutrition transition in sub-Saharan Africa showed that the main causes of overweight and obesity included lifestyle changes, such as physical inactivity, and increased energy, fat, and sugar intake, in addition to other sociodemographic factors [9]. It is important to address the double burden simultaneously through the promotion of diversified, balanced, and healthy diets, including adequate amounts of high-quality protein and fat, plenty of vegetables and fruits, and moderate amounts of sugar and salts [10,11].

In 1998, a joint World Health Organization (WHO) and Food and Agriculture Organization consultation issued guidelines for developing and using food-based dietary guidelines (FBDGs) [12], which are easier to put into practice than nutrient-based guidelines. These were intended to contribute to health by providing suggestions regarding the appropriate quality and quantity of food to be consumed in order to avoid nutrient excess or deficiency. Additionally, it is important that these guidelines consist of readily available ingredients, are easy to understand and accepted tools, and are very feasible to implement. In Japan, individuals with higher adherence to the Japanese dietary guidelines had a lower risk of total mortality [13]. In the United States, a higher Healthy Eating Index, indicating adherence to the Dietary Guidelines for Americans and Alternative Healthy Eating Index, was associated with a significant reduction in the risk of all-cause mortality, major chronic diseases [14], and genetic weight gain [15]. Thus, diets that follow country-specific FBDGs are required in order to promote healthy eating habits among their citizens and, consequently, reduce overall disease risk. In 2010, the *Kenya National Clinical Nutrition Reference Manual* [16] proposed the Food Pyramid (FP) for Kenyan people (Supplementary Figure S1), which is a visual aid indicating the recommended number of servings (SVs) of each food group. The FP was further modified in the *National Guidelines for Healthy Diets and Physical Activity* in 2017 [11]. The Kenyan FP is expected to be utilised for dietary evaluation and nutritional education in order to prevent the double burden of malnutrition by guiding ideal diets for Kenyans, providing adequate energy and nutrient intake, and supporting a healthy weight status and positive health outcomes.

Although the FP is used in Kenya, it has challenges. Firstly, it is not clear whether the recommended number of servings for each food group (based on the US Food Pyramid of 1992) provides adequate nutrient intake. Secondly, to the best of our knowledge, no previous study has evaluated the adherence to the Kenyan FP’s recommendations. This study aimed to investigate the adherence to the recommendations provided in the FP, and whether higher adherence can avoid excess or insufficient energy and nutrient intake. This study evaluated the level of adherence based on the recommended amounts for each food group as shown in the Kenyan FP, following the method prescribed by Kurotani et al. [13].

## 2. Materials and Methods

### 2.1. Participants

This cross-sectional survey was originally conducted to identify factors related to normal BMI in Wangige Village, Kiambu County, located to the north-west of Nairobi—the capital city of Kenya. This county is home to mainly the Kikuyu people—a major ethnic group in Kenya—and is the area with the second largest population in Kenya (approximately 2.42 million people), following the city of Nairobi [17]. Agriculture is the primary economic activity in the county, and contributes to 17% of the county’s population income. The county’s average rainfall is 1200 mm, and the average temperature is 26°C. Approximately 95% of the total population is literate [18]. Wangige Village is located in Kabete Sub-county—the most densely populated area in the county, due to its proximity to the capital city—and has a large market called Wangige Market [17]. This area was chosen because of its mixed rural and urban population, which might be in the process of urbanisation and nutrition transition, and because of its accessibility from Nairobi. According to the 2014 Demographic Health Survey, the percentage of overweight and obese adult women in Kiambu County was 45.7%, which was higher than the national level of 32.8% [6].

Participants were married couples from farming households between 18 and 60 years of age living in Wangige Village. Target households were randomly selected if they were engaged in agriculture and both spouses were at home. After the researcher (K.H.) explained the purpose of the study in the local language, and with the local staff’s cooperation, men and women from the households who provided consent were included in the study. Pregnant and lactating women were not included. Of 148 male and female participants from 72 households who took the survey, we excluded a total of 28 participants: 5 for missing BMI data, 1 BMI outlier (BMI 66 kg/m^2^), and 22 for missing dietary data. The reasons for missing dietary data were declining participation after filling out the consent form and not being home at the time of the survey. We finally analysed data from 56 men and 64 women across 64 households.

This study was conducted in accordance with the guidelines laid down in the Declaration of Helsinki, and all procedures involving research study participants were approved by the KNH-UoN Ethics and Research Committee of the University of Nairobi (approval number: KNH-ERC/A/129; approval date: 5 April 2019).

### 2.2. Study Design

This cross-sectional study was conducted during the short rainy season, October–November 2019. All of the surveys were conducted over three days during weekdays. On the first day, data on demographic and physical characteristics were collected. On the second and third days, 24-h dietary recall interviews were conducted. Two local staff members visited each household and completed all of the surveys, and during each visit the researcher (K.H.) confirmed that there were no omissions or mistakes. The local staff received training in collecting physical measurements through a 2-day course provided by nurses at Kenyatta National Hospital, and received 24-h dietary recall training in advance by the researcher (K.H.) in order to ensure that there were no errors in the dietary survey.

### 2.3. Demographic Survey

The local staff interviewed participants about their sex, age, household size, level of education, and socioeconomic status (SES), and collected their responses in the “KoBo Toolbox”—a tool for field data collection developed by the Harvard Humanitarian Initiative [19]. Household size was defined as the number of people sharing the same kitchen. Levels of education were categorised as follows: none, primary school level (those who graduated pre-primary and primary school), secondary school level, and university level (those who graduated polytechnic university and college/university). We used the WFP’s creation of wealth index [20] as a reference to calculate SES. We conducted a principal component analysis using monthly household income, access to water, presence of toilet facilities, groupings of the number of each asset owned (2–3 or 4–7), and preferred heat source for cooking (presence of gas or biogas) as variables to create the new SES variable. The SES variable was categorised equally into three groups: low, middle, and high. The respondents’ number of assets was determined by whether they owned cell phones, bicycles, motorcycles, cars, radios, televisions, and refrigerators.

### 2.4. Physical Measurements

To assess the health status and energy balance of the participants, physical measurements were carried out on items of particular interest [4]. The participants’ height and waist–hip circumference were measured with a measuring tape, and their weight was measured with a scale (HD-660, Tanita Co., Tokyo, Japan). The BMI was calculated using the formula of weight (kg) divided by height squared (m^2^), and was categorised as follows: underweight for BMI < 18.5, normal weight for BMI 18.5–24.9, overweight for BMI 25.0–29.9, and obese for BMI > 30.0 [21]. Body fat percentage was measured using a handheld body fat monitor (HBF-306C, Omron Co., Tokyo, Japan). Blood pressure was measured using an upper-arm blood pressure monitor (HEM-7130-HP, Omron Co., Tokyo, Japan). Hypertensive individuals were those with systolic blood pressure (SBP) of 140 mmHg or higher and/or diastolic blood pressure (DBP) of 90 mmHg or higher, while those with severe hypertension were those with SBP of 160 mmHg or higher and/or DBP of 100 mmHg or higher [4]. As a proxy indicator of physical activity, step counts were measured using a wearable monitor over three consecutive dates, and average daily step counts were calculated (Fitbit Surge, Fitbit, CA, USA).

### 2.5. Dietary Survey

Dietary intake was assessed using 24-h dietary recalls for two consecutive days using a food atlas [22]. Trained staff interviewed each participant about the dishes, ingredients, and drinks they consumed in the past 24 h. To estimate each dish’s amount, the staff presented the participants with pictures of foods and asked them to select a picture reflecting the nearest quantity that they consumed from a food atlas [22]. The food atlas contains 173 food and dish items with 1–3 different sizes of pictures for each dish; it was developed to evaluate the food sizes of Kenyan adolescents in an urban setting. These pictures were used as a reference for estimating the amount of food consumed. The energy and nutrient intakes were computed from estimated quantities of foods consumed using the Kenyan Food Composition Tables (KFCT) 2018 [23], including 522 food items and 142 cooked dish items. Since soft drinks (soda), juice, and coffee did not have similar food data in the KFCT 2018, the 2015 edition of the Standard Tables of Food Composition in Japan (7th revision) [24] was used for those items. In this study, the average daily intake for two days was calculated.

For 0.6% of all items, the intake amount values were missing. The missing values were compensated for by the similar dish/food’s average intake by the same sex. For 10% of the total participants who completed the dietary survey on only one day, the one-day intake was used instead of the average of the two days.

The number and percentage of people at risk of excess or insufficient nutrient intakes were calculated based on whether an individual’s average intake was higher or lower than the age- and sex-specific reference level for each nutrient [25] (Supplementary Table S1). Energy intake was assessed using individual’s weight status as defined by BMI. Macronutrients were assessed by calculating energy ratios and using the upper and lower limits of acceptable macronutrient distribution ranges. Protein intake per body mass and 11 micronutrient intakes were compared to estimated average requirements (EARs) to assess the risk of insufficiency. The risk of insufficiency for total fibre and calcium were assessed using adequate intake because EARs were not shown. The risk of excess for sodium and insufficiency of potassium were assessed based on the WHO’s recommendations [26,27].

### 2.6. Adherence to the Kenyan Food Pyramid’s Recommendations

In the Kenyan dietary guidelines, 1 SV is defined as dry weight for general starches (cereals, grains, roots, and tubers), volume for milk products and plant-based foods, and raw weight for other groups; examples of 1 SV size were shown only for major food items by number of cups or teaspoons [11,16] (Supplementary Table S2). To compute the total number of SVs, we first defined 1 SV size according to the main nutrient content (Table 1). Next, the serving sizes of frequently consumed dishes in the study were determined. Then, the number of SVs for each food group and sub-category were calculated. The general starchy group was divided into “cereals and grains” and “roots and tubers”. Pulses and legumes were called “plant-based foods”, while meat, fish, chicken, and eggs were called “animal-based foods”. The sum of “plant-based foods” and “animal-based foods” was calculated as the protein-rich food group. The vegetable group was divided into green leafy vegetables and other vegetables. The definition of green leafy vegetables was in accordance with the classification of the minimum dietary diversity for women [28]. The SVs of the oil and sugar groups were counted only for dishes that were frequently consumed and contain a lot of fat and sugar based on Kenyan food recipes [24,29].

The recommended SVs were described according to the three levels of physical activity: lower, moderate, and higher [11,16]. The recommendations of the grain group and meat group were applied to the general starchy group and protein-rich food group, respectively. The recommended SVs of oils and sugar were not shown. Because physical activity levels were not measured in this study, the minimum and maximum of recommended SVs from low to high physical activity levels were used as the recommended range for all participants. For scoring, 10 points (maximum) were given if the intake amount was in the recommended range. Since the WHO does not set an upper limit for vegetables [10], we did not set an upper limit for the number of SVs either, and the maximum score was 10 if the intake was 3 SVs or more. If the intake amount was outside the recommended range, the score was calculated using Equation (1) or (2) to make the score less than 10 points, according to the scoring method developed in the study by Oba et al. [30].

If the number of SVs was below the recommended range:    10 × (number of SVs/lower limit of the recommended range).(1)

If the number of SVs exceeded the recommended range: 10 − 10 × (number of SVs − upper limit)/upper limit. (2)

The degree of adherence to the recommended SV ranges in the FP was assessed by the sum of the scores for general starches, milk products, protein-rich foods, and vegetables and fruits, each with a maximum of 10 points (maximum of 50 points) (hereafter, “FP score”).

### 2.7. Statistical Analyses

The participants were divided into tertiles (low, middle, and high) by the FP scores. Nutrient intake and the number of SV counts from each food group were energy-adjusted using the residual method [31]. The association of FP scores with demographic characteristics, energy and nutrient intake, number of SV counts from each food group, and the number of those who were at risk of excess or insufficiency for each nutrient were evaluated using the Mantel–Haenszel-test for categorical variables and the Jonckheere–Terpstra test for continuous variables. All statistical analyses were conducted using IBM SPSS Statistics ver. 23 (IBM, New York, NY, USA), and the significance level was set at 5% for a two-tailed test.

## 3. Results

### 3.1. Characteristics of Participants

The average FP score was 25.0, with minimum and maximum scores of 14.1 and 41.5, respectively. There was no significant difference in FP scores between men and women (24.5 ± 4.8 for men, 25.4 ± 5.6 for women) (*p* = 0.400). After dividing the FP scores into tertiles, the average and standard deviation of scores for each group were 19.4 ± 2.2, 24.4 ± 1.3, and 31.0 ± 3.1 for the low, middle, and high groups, respectively.

Table 2 shows the demographic characteristics by the FP score tertiles. The higher FP score group tended to include more participants engaged in full-time farming—there was no association between the FP score and SES. BMI, waist and hip circumference, body fat, blood pressure, and step counts did not differ significantly between the FP score tertiles. The higher FP score group had a significantly higher meal frequency (*p* for trend = 0.010).

### 3.2. Energy and Nutrient Intake, and SV Counts

Table 3 shows energy or energy-adjusted nutrient intakes by FP score tertiles. The higher FP score group had significantly more energy intake. The higher FP score group also consumed more energy-adjusted protein, fibre, sodium, potassium, calcium, magnesium, phosphorous, iron, zinc, selenium, vitamin A, and vitamin C.

Table 4 shows the SV counts from each food group by the FP score tertiles. The higher FP score group consumed significantly fewer SVs from cereals and oils, and more SVs from milk products, animal-based foods, green leafy vegetables, and sugars.

**Figure 1:**
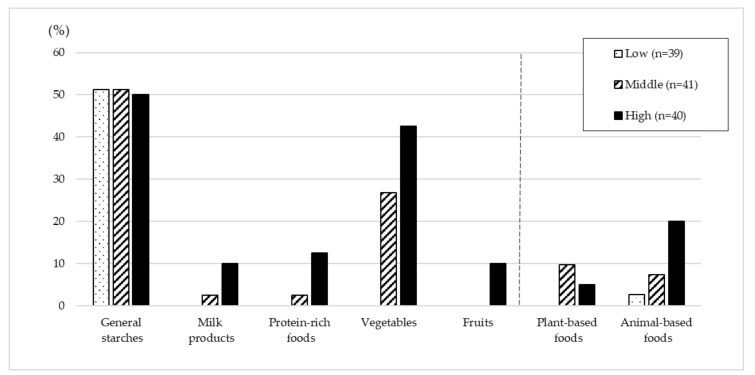
The proportion of those who met the recommendations by FP score* tertiles. * FP score indicates adherence to the recommendations of the Kenyan FP, and was calculated from the individual average dietary intake over two days.

Figure 1 shows the proportion of participants within the recommended SV ranges for each food group by the FP score tertiles. In all three groups, approximately 50% of the participants were in the recommended range for the general starches group. In the lowest FP score group, there were no participants in the recommended ranges for milk and milk products, protein-rich foods, vegetables, or fruits. Even with the highest FP score group, less than 15% were in the recommended range for milk and milk products, protein-rich foods, and fruits.

### 3.3. Risk of Excess or Insufficient Nutrient Intake against Recommended Nutrient Intakes

Table 5 shows the proportion of participants at risk of excess or insufficient intake for each nutrient by the FP score tertiles. In all of the FP score groups, more than half of the participants did not meet the EARs for magnesium, selenium, vitamin A, vitamin B1, and vitamin B2. The higher FP score group had a significantly lower risk of insufficiency for protein per body mass (kg), protein–energy ratio, total fibre, calcium, magnesium, zinc, selenium, vitamin A, vitamin B2, vitamin B12, folic acid, and vitamin C. Conversely, the higher FP score group had a significantly higher risk of excessive sodium intake.

## 4. Discussion

The present study suggests that the higher FP score groups—i.e., those with higher adherence to the Kenyan FP—can avoid the risk of insufficient nutrient intake. However, they cannot avoid the risk of excess energy and sodium intake. The Kenyan FP can at least be a useful dietary evaluation tool to avoid nutrient deficiency. This study is the first to examine the association between adherence to the Kenyan FP and the adequacy of nutritional intake.

The strength of this study was the standardisation of survey skills among staff for data collection. All of the staff members received the same training, and the researcher (K.H.) checked for any unnatural data or missing items in each survey. Since the food intake was estimated using a food atlas [22], the error caused by differences in the perceptions of the participants and staff members regarding the actual intake amounts was likely minimised [32]. Additionally, the serving sizes for frequently consumed dishes were defined so as to easily calculate the number of SVs. In addition, we collected data on dietary intake, as well as physical characteristics such as BMI and body fat percentage; this allowed us to assess whether the participants’ energy intake was excessive or deficient.

Higher adherence to the FP showed greater food variety and, simultaneously, higher intake of micronutrients. The higher adherence group consumed a smaller proportion of staple foods and more vegetables and protein-rich foods. These results indicate an association between consuming various foods and intake of required nutrients [33], suggesting that the FP helps to avoid the risk of inadequate micronutrient intake by encouraging a balanced intake of various foods, rather than mainly consuming staple foods.

We could not assess the absolute intake of B vitamins because of the lack of information about fortified products. Fortification of cereals such as maize and wheat flour with B vitamins has been mandatory in Kenya since 2017 [34]. Hence, we calculated the intake of B vitamins based on the assumption that all participants consumed commercially fortified flour. However, Khamila et al. previously reported that compliance with national standards for fortified maize flour was low, with only 11.1% of the samples complying with regulations [35]. Generally, foods rich in B vitamins mainly include whole grains, animal-based foods, and peas [24]. A still high risk of vitamin B1 and niacin insufficiency in the high FP score group may be explainable by the fact that the higher FP score groups consumed fewer cereals and had an insufficient intake of animal-based foods. Even in the highest FP score group, only 20% consumed the recommended amount of animal-based foods. B vitamin sources may differ depending on whether fortified foods are consumed, so future dietary surveys need to confirm the use of fortified flour.

The FP could not assess excess intakes of energy and sodium. Our results show that higher adherence to the FP also correlated with higher energy, sodium, and sugar intakes. Higher intakes of energy and sugar may be caused by the number of meals and habitual intake of milk tea with sugar. The average number of meals was more than three in all FP score groups. Almost all of the participants consumed milk tea with sugar several times a day as breakfast and as a snack. In addition, participants in the high FP score group had milk tea more often than those in other groups—in fact, they also consumed significantly more milk products than others. Savy et al. reported that high snacking frequency was associated with high dietary diversity in a study of urban areas in Burkina Faso [36]. Higher sodium intake may be related to the greater variety of foods in the higher FP score group. Thus, it is not surprising that higher consumption of vegetables and animal products indicates higher sodium intake in the form of seasoning. In the present study, we estimated the amount of seasoning used from common recipes [29]. Although actual oil and seasoning use in dishes are unclear, subjects in the higher FP score group consumed more seasonings because it is difficult to consume a large variety of foods with fewer seasonings. The WHO recommends limiting the intake of sugar and salt as part of a healthy diet [10]. Previous studies on food-based dietary guidelines contain information on excessive consumption of total energy, sugar-sweetened drinks, fruit juice, snacks, and alcoholic beverages, and have demonstrated the effectiveness of the guidelines on health outcomes [13,14,15]. The current FP may be improved by including such information on refraining from excessive energy, salt, and sugar intake in order to prevent NCDs.

The Kenyan FP, established based on the US FP, should be modified based on local dietary patterns. The average adherence to the FP was approximately 50% of the maximum FP score in this study. Even in the highest FP score group, only ~10% consumed the recommended SVs for milk products, protein-rich foods, and fruits, suggesting limited access to these foods, consumption habits, or other factors in the study area. Darmon et al. reported in their review that nutritious foods such as whole grains, lean meat, fish, low-fat dairy products, and fresh vegetables and fruits were more likely to be consumed by individuals with higher SES [37]. However, there was no significant association between FP score and SES in the present study. The low adherence to the FP’s recommendations may be due to the inaccessibility of foods and the unsuitable recommended ranges of each food group. Food groups and the recommended range of each food group should be set after considering the availability of foods, food culture, and the target population’s food environment, in addition to the recommended nutrient intake. Sarfo et al. used a linear programming approach to determine nutritious diets for women and children in rural Kenya, considering food availability and cost; they estimated that the inclusion of three traditional vegetables would provide high levels of micronutrients without further increasing cost [38]. Promoting home-grown traditional vegetables and other crops would improve access to nutritious foods even for low-income households.

This study had some limitations. Firstly, we did not consider the sex-based differences in dietary habits because our goal was to examine the association between adherence to the FP and average nutrient intake. Secondly, there is a possibility that the number of days in the dietary survey was not sufficient to evaluate the habitual risk of insufficient nutrient intake. In this study, a 24-h recall dietary survey was only conducted on two consecutive days during the rainy season, due to manpower constraints. To evaluate the habitual risk of inadequate nutrition, conducting the survey on non-consecutive days—including holidays in both the rainy and dry seasons—is necessary. Thirdly, as mentioned above, nutrient intakes were calculated based on the types and amounts of cooking oils and seasonings indicated in standard recipes for common Kenyan home cooking [29]. Therefore, the individual differences in the amounts of cooking oils and seasonings were not considered. In addition, as we could not identify whether or not the participants consumed fortified flour, the actual intake of B vitamins could not be assessed. The calculated intake of B vitamins may have been overestimated, and the risk of insufficiency may have been underestimated because we assumed that all participants consumed fortified flour; this is an important point to consider in future dietary surveys. Fourthly, since the participants were limited to married couples, it was possible that the couples had similar diets and were classified in the same FP score group; this may have had some effect on the statistical power. Finally, the sample size was small, and only farmers from a single region were included. We believe that this sample was representative of the general population, at least in this area, but it is not clear whether the results of this study can be applied to populations in other regions. Further studies may be needed for regions or ethnic groups with significantly different diets and food environments, such as urban people or pastoralists; however, since agriculture is the primary industry, and the Kikuyu people are the most abundant ethnic group in Kenya, the findings of this study will apply to many Kenyans.

## 5. Conclusions 

Dietary evaluation using the Food Pyramid was an effective tool to avoid the risk of insufficient nutrient intake. However, the current Food Pyramid may not be effective for avoiding excessive energy, salt, and sugar intake. In future, it will be necessary to include evaluations to limit energy, sugar, and salt. Food groups and the recommended range of the Food Pyramid should be optimised according to the dietary environment of the target population for the promotion of their health. 

## Figures and Tables

**Table 1:** Definition of 1 SV and the FP score criteria for each food group.

Food Group ^(1)^	Definition of 1 SV ^(2)^	Recommended SVs ^(3)^ (SVs/Day)	FP Score
General starches	20 g carbohydrate	6–11	0–10
Cereals and grains			
Roots and tubers			
Milk products	300 mg Ca	2–3	0–10
Protein-rich foods	6 g protein	5–7	0–10
Plant-based foods			
Animal-based foods			
Vegetables	80 g edible weight	≧3	0–10
Green leafy vegetables			
Other vegetables			
Fruits	100 g edible weight	2–3	0–10
Total			0–50

FP: Food Pyramid; SV: serving; Ca: Calcium, ^(1)^ The number of SVs was also calculated in sub-categories to examine the quality of the diet. ^(2)^ Defined in the present study. ^(3)^ Defined by the *Kenya National Clinical Nutrition and Dietetics Reference Manual, 2010*.

**Table 2:** Characteristics of participants.

	FP Score Tertiles			*p* *
	Low (*n* = 39)	Middle (*n* = 41)	High (*n* = 40)	
FP score	19.4 ± 2.2	24.4 ± 1.3	31.0 ± 3.1	-
Age (years old)	42.5 ± 10.2	41.8 ± 9.2	43.1 ± 9.4	0.830
Gender (women %)	19 (48.7)	23 (56.1)	22 (55.0)	0.580
Household size (person)	4.6 ± 1.3	4.9 ± 1.5	4.5 ± 1.4	0.548
Full-time farmer	31 (79.5)	32 (78.0)	38 (95.0)	0.059
Education level				
Primary school	14 (35.9)	12 (29.3)	13 (32.5)	0.987
Secondary school	15 (38.5)	22 (53.7)	18 (45.0)	
University	10 (25.6)	7 (17.1)	9 (22.5)	
Socioeconomic status				
Low	15 (38.5)	15 (36.6)	9 (22.5)	0.660
Middle	11 (28.2)	13 (31.7)	21 (52.5)	
High	12 (30.8)	13 (31.7)	9 (22.5)	
BMI (kg/m^2^)	25.8 ± 5.2	25.5 ± 5.2	26.7 ± 5.1	0.284
Underweight	2 (5.1)	2 (4.9)	3 (7.5)	0.344
Normal weight	18 (46.2)	18 (43.9)	13 (32.5)	
Overweight	12 (30.8)	14 (34.1)	13 (32.5)	
Obesity	7 (17.9)	7 (17.1)	11 (27.5)	
Waist circumference (cm)	92.4 ± 11.9	90.8 ± 16.2	94.8 ± 13.9	0.426
Hip circumference (cm)	105.8 ± 13.0	103.8 ± 12.3	105.9 ± 9.7	0.745
W/H ratio	0.88 ± 0.07	0.87 ± 0.08	0.89 ± 0.09	0.182
Body fat (%)	28.4 ± 8.0	28.1 ± 7.1	29.3 ± 8.7	0.641
SBP(mmHg)	129.6 ± 20.9	129.9 ± 13.1	131.1 ± 19.3	0.392
DBP(mmHg)	83.6 ± 11.5	83.9 ± 13.5	83.7 ± 11.2	0.881
Hypertension	12 (30.8)	13 (31.7)	16 (40.0)	0.387
Severe hypertension	5 (12.8)	4 (9.8)	3 (7.5)	0.433
Step counts (steps/day)	13,580 ± 9258	14,747 ± 8539	14,955 ± 8011	0.291
Meal frequency (times/day)	3.4 ± 0.9	3.6 ± 0.8	4.1 ± 1.2	0.010

Values are the mean ± standard deviation or the number (percentage) of participants. FP: Food Pyramid; SBP: systolic blood pressure; DBP: diastolic blood pressure; * *p*-values are based on Mantel–Haenszel chi-squared for categorical variables and the Jonckheere–Terpstra test for continuous variables.

**Table 3:** Relationship between energy or nutrient intake and FP score tertiles.

Energy and Nutrient Intakes	FP Score Tertiles			*p* for Trend *
	Low (*n* = 39)	Middle (*n* = 41)	High (*n* = 40)	
Energy (kcal)	1721 ± 817	1793 ± 665	1976 ± 514	0.002
Protein (%E)	11.5 ± 2.8	11.7 ± 1.8	12.4 ± 2.0	0.006
Fat (%E)	26.5 ± 4.1	26.5 ± 6.3	27.3 ± 5.1	0.584
Carbohydrate (%E)	62.0 ± 4.6	61.8 ± 6.1	60.3 ± 4.9	0.152
Protein (g)	50.2 ± 11.1	52.2 ± 10.1	56.1 ± 9.7	0.001
Fat (g)	56.4 ± 8.0	55.7 ± 11.7	55.5 ± 13.6	0.260
Carbohydrate (g)	263.3 ± 19.3	260.6 ± 26.7	256.6 ± 28.2	0.361
Fibre (g)	26.7 ± 9.3	31.4 ± 9.2	33.8 ± 8.9	<0.001
Sodium (mg)	1804 ± 757	1921 ± 584	2136 ± 871	0.028
Potassium (mg)	1913 ± 803	2146 ± 589	2266 ± 599	0.002
Calcium (mg)	641 ± 165	788 ± 273	869 ± 274	<0.001
Magnesium (mg)	257 ± 62	296 ± 62	328 ± 64	<0.001
Phosphorous (mg)	1415 ± 262	1491 ± 281	1556 ± 305	0.018
Iron (mg)	18.0 ± 8.3	19.6 ± 7.2	19.8 ± 5.3	0.031
Zinc (mg)	8.0 ± 2.5	8.3 ± 2.1	9.2 ± 1.9	<0.001
Selenium (µg)	34.3 ± 17.7	40.5 ± 18.7	46.0 ± 19.7	0.002
Vitamin A (µgRAE)	280 ± 101	370 ± 191	426 ± 160	<0.001
Vitamin B_1_ (mg)	1.09 ± 0.37	1.09 ± 0.28	0.98 ± 0.31	0.117
Vitamin B_2_ (mg)	0.93 ± 0.23	1.03 ± 0.37	0.95 ± 0.29	0.825
Niacin (mg)	11.4 ± 3.1	11.5 ± 1.7	11.8 ± 2.8	0.355
Vitamin B_12_ (µg)	2.2 ± 0.8	2.2 ± 1.0	2.5 ± 1.1	0.171
Folic acid (µg)	487 ± 204	529 ± 141	451 ± 166	0.227
Vitamin C (mg)	71 ± 49	94 ± 59	118 ± 62	<0.001

Values are the mean ± standard deviation, and are energy-adjusted. FP: Food Pyramid; RAE: retinol active equivalent; * *p*-values are based on the Jonckheere–Terpstra test.

**Table 4:** Relationship between the food group SV counts and FP score tertiles.

Food Group Intakes (g)	FP Score Tertiles			*p* for Trend *
	Low (*n* = 39)	Middle (*n* = 41)	High (*n* = 40)	
General starches	10.73 ± 1.81	10.16 ± 1.47	9.10 ± 1.60	<0.001
Cereal and grains	9.28 ± 1.76	9.18 ± 1.56	8.23 ± 1.60	0.007
Roots and tubers	1.44 ± 2.36	0.98 ± 1.22	0.87 ± 1.41	0.084
Milk products	0.92 ± 0.32	0.91 ± 0.36	1.28 ± 0.49	0.001
Protein-rich foods	1.62 ± 1.95	2.08 ± 1.95	2.95 ± 2.03	0.001
Plant-based foods	0.82 ± 0.96	1.05 ± 1.05	1.19 ± 1.52	0.380
Animal-based foods	0.80 ± 1.77	1.03 ± 1.83	1.76 ± 1.99	0.022
Meats	0.68 ± 1.64	0.78 ± 1.79	1.46 ± 1.84	0.126
Fishes	0.12 ± 0.54	0.01 ± 0.03	0.01 ± 0.02	0.263
Eggs	0.06 ± 0.24	0.25 ± 0.61	0.30 ± 0.65	0.339
Vegetables	1.37 ± 0.91	2.67 ± 1.88	2.58 ± 1.27	<0.001
Green leafy vegetables	0.95 ± 0.75	1.86 ± 1.79	1.97 ± 1.26	<0.001
Other vegetables	0.42 ± 0.51	0.81 ± 0.73	0.61 ± 0.83	0.694
Fruits	0.05 ± 0.12	0.05 ± 0.17	0.45 ± 0.97	0.725
Oils	5.78 ± 5.35	4.63 ± 4.33	2.65 ± 3.75	<0.001
Sugars	3.80 ± 1.34	3.66 ± 1.45	5.08 ± 1.98	0.003

Values are the mean ± standard deviation, and are energy-adjusted. FP: Food Pyramid; SV: serving; * *p*-values are based on the Jonckheere–Terpstra test.

**Table 5:** The risk rate of insufficient or excess nutrient intake by the FP score tertiles.

	Criteria for Risk of Insufficient or Excess ^(1)^	FP Score Tertiles			*p* for Trend *
		Low (*n* = 39)	Middle (*n* = 41)	High (*n* = 40)	
Energy (insufficient)	Underweight	1 (2.6)	2 (4.9)	3 (7.5)	0.316
Energy (excess)	Overweight and obese	19 (48.7)	21 (51.2)	24 (60.0)	0.316
Protein per kg BM (insufficient)	EAR	21 (53.8)	17 (41.5)	11 (27.5)	0.018
Protein %E (insufficient)	LL of AMDR	15 (38.5)	5 (12.2)	4 (10.0)	0.002
Fat %E (insufficient)	LL of AMDR	2 (5.1)	6 (14.6)	3 (7.5)	0.724
Fat %E (excess)	UL of AMDR	1 (2.6)	6 (14.6)	3 (7.5)	0.437
Carbohydrate %E (excess)	UL of AMDR	11 (28.2)	12 (29.3)	6 (15.0)	0.170
Total fibre (insufficient)	AI	17 (43.6)	10 (24.4)	8 (20.0)	0.022
Sodium (excess)	WHO recommendation	10 (25.6)	16 (39.0)	22 (55.0)	0.008
Potassium (insufficient)	WHO recommendation	36 (92.3)	37 (90.2)	37 (92.5)	0.973
Calcium (insufficient)	AI	35 (89.7)	33 (80.5)	26 (65.0)	0.008
Magnesium (insufficient)	EAR	29 (74.4)	24 (58.5)	15 (37.5)	0.001
Iron (insufficient)	EAR	3 (7.7)	1 (2.4)	0 (0.0)	0.058
Zinc (insufficient)	EAR	28 (71.8)	21 (51.2)	8 (20.0)	<0.001
Selenium (insufficient)	EAR	32 (82.1)	30 (73.2)	19 (47.5)	0.001
Vitamin A (insufficient)	EAR	38 (97.4)	36 (87.8)	28 (70.0)	0.001
Vitamin B1 (insufficient)	EAR	24 (61.5)	23 (56.1)	19 (47.5)	0.211
Vitamin B2 (insufficient)	EAR	29 (74.4)	25 (61.0)	19 (47.5)	0.015
Niacin (insufficient)	EAR	17 (43.6)	18 (43.9)	18 (45.0)	0.900
Vitamin B12 (insufficient)	EAR	22 (56.4)	24 (58.5)	10 (25.0)	0.005
Folic acid (insufficient)	EAR	23 (59.0)	13 (31.7)	14 (35.0)	0.032
Vitamin C (insufficient)	EAR	22 (56.4)	19 (46.3)	10 (25.0)	0.005

Values are the number (percentage) of participants. FP: Food Pyramid; BM: body mass; %E: % energy; EAR: estimated average requirement; LL: lower limit; UL: upper limit; AMDR: acceptable macronutrient distribution ranges; AI: adequate intake; ^(1)^ Institute of Medicine (2006) and World Health Organization (2012); * *p*-values are based on the Mantel–Haenszel chi-squared test.

## Data Availability

The datasets used and/or analysed during the current study are available from the corresponding author on reasonable request.

## Supplementary Materials

The following are available online at https://www.mdpi.com/article/10.3390/nu13124470/s1: Figure S1: Kenyan Food Pyramid; Table S1: Reference values for the risk assessment of nutrient insufficiency and excess; Table S2: Serving sizes of foods in each food group.
