# Supplementary material for: High Adherence to the Food Pyramid’s Recommendations Avoids the Risk of Insufficient Nutrient Intake among Farmers in Peri-Urban Kenya
Source: Nutrients. 2021 Dec 14;13(12):4470. doi: 10.3390/nu13124470 (PMC8707577; doi:10.3390/nu13124470)
Supplement: Supplementary file 1 [file nutrients-13-04470-s001.zip › nutrients-1457164-supplementary.pdf]

Supplemental figure and tables

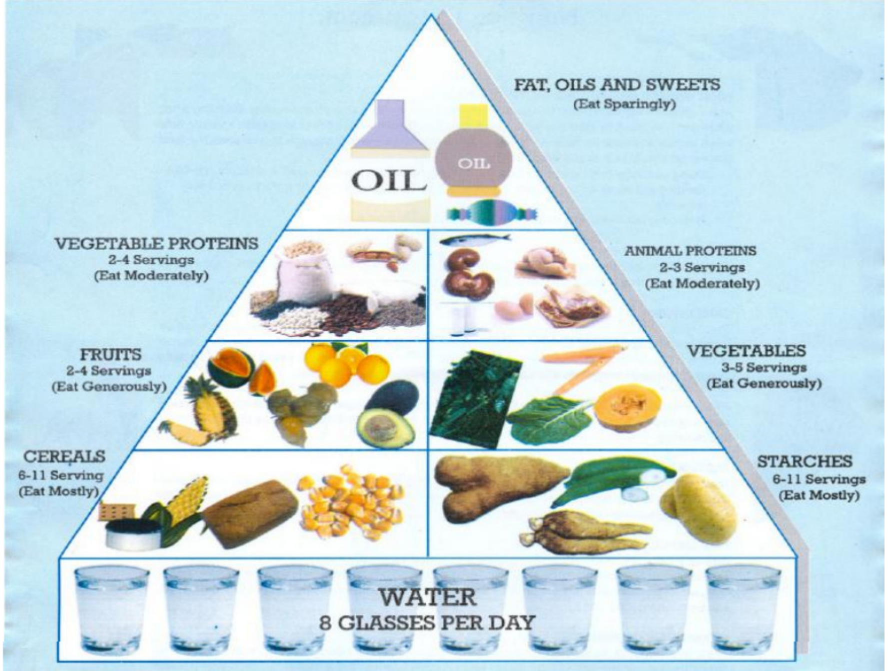

Figure S1. Kenyan Food Pyramid.

Source: *Kenya National Clinical Nutrition and Dietetics Reference Manual* (2010), Ministry of Medical services, Kenya

Table S1. Reference value for the risk assessment of nutrient insufficiency and excess.

	Criteria for risk of insufficient or excess [1)]	Reference value [1)]	
		Men	Women
Energy (insufficient)	Underweight	BMI < 18.5 kg/m^2	
Energy (excess)	Overweight and obese	BMI ≧ 25.0 kg/m^2	
Protein per kg BM (insufficient)	EAR	0.66 g per kg BM	
Protein %E (insufficient)	Lower limit of AMDR	10 %	
Protein %E (excess)	Upper limit of AMDR	35 %	
Fat %E (insufficient)	Lower limit of AMDR	20 %	
Fat %E (excess)	Upper limit of AMDR	35 %	
Carbohydrate %E (insufficient)	Lower limit of AMDR	45 %	
Carbohydrate %E (excess)	Upper limit of AMDR	65 %	
Total fiber (insufficient)	AI	14 g/1000 kcal	
Sodium (excess)	WHO recommendation	2000 mg	
Potassium (insufficient)	WHO recommendation	3510 mg	
Calcium (insufficient)	AI	1000 mg	
Magnesium (insufficient)	EAR	330 mg for 19-30y<br>350 mg for over 31y	255 mg for 19-30y<br>265 mg for over 31y
Iron (insufficient)	EAR	6.0 mg	8.1 mg for 19-50y<br>5.0 mg for over 51y
Zinc (insufficient)	EAR	9.4 mg	6.8 mg
Selenium (insufficient)	EAR	45 μg	
Vitamin A (insufficient)	EAR	625 μg RAE	500 μg RAE
Vitamin B1 (insufficient)	EAR	1.0 mg	0.9 mg
Vitamin B2 (insufficient)	EAR	1.1 mg	0.9 mg
Niacin (insufficient)	EAR	12 mg	11 mg
Vitamin B12 (insufficient)	EAR	2.0 μg	
Folic acid (insufficient)	EAR	320 μg	
Vitamin C (insufficient)	EAR	75 mg	60 mg

FP, food pyramid; BM, Body mass; %E, % energy; EAR, Estimated Average Requirement; AMDR, Acceptable Macronutrient Distribution Ranges; AI, Adequate Intake; [1)] Institute of Medicine (2006) and World Health Organization (2012).

Table S2. Serving size of foods in each food group by Kenyan government.

Food group	Equivalent amount weight / volume [1)]	Dish item [2)]	Equivalent amount for 1 SV	Main nutrient content or weight
**General starches**	**30 g dry weight**			**20 g Carb**
Cereals and grains		Ugali	75 g	20 g Carb
		Cooked Rice	75 g	20 g Carb
		Chapati	1/2 slice, 1 slice = 80 g	20 g Carb
		Pancake	50 g	
		Cooked pasta	75 g	18 g Carb
		Bread	50 g (2 slices)	24 g Carb
		Porridge	300 ml	26 g Carb
		Githeri (maize & beans)	maize 100 g	21 g Carb
		Mandazi (doughnut)	50 g (1 piece)	24 g Carb
		Cup cake	50 g (1 piece)	24 g Carb
Roots and tubers		Arrowroot	100 g	22 g Carb
		Sweet potatoes	100 g	21 g Carb
		Irish potatoes	100 g	23 g Carb
		French fried potato	50 g	22 g Carb
		Cassava	60 g	20 g Carb
		Cooked banana (plantain)	100 g	20 g Carb
**Milk products**	**250 ml**			**300 mg Ca**
		Milk	250 ml	300 mg Ca
		Milk tea	2 cups (Half the amount of milk tea)	286 mg Ca
		Yoghurt	200 ml	300 mg Ca
**Pulses (beans, peas, lentils) (Plant-based foods)**	**125 ml**			**6 g Protein**
		Cooked soybeans	50 g (1/3 cup)	7.0 g Protein
		Cooked cowpeas	70 g (1/2 cup)	6.7 g Protein
		Cooked lentils	70 g (1/2 cup)	7.5 g Protein
		Cooked green grams	70 g (1/2 cup)	7.1 g Protein
		Cooked pigeon peas	70 g (1/2 cup)	6.4 g Protein
		Dried groundnuts	30 g	6.0 g Protein

(*to be continued*)

Table S2. Cont.

**Meat, fish, chicken, eggs (Animal-based foods)**	**30 g**			**6 g Protein**
		Nile tilapia	30 g	5.9 g Protein
		Stewed chicken	30 g	5.6 g Protein
		Stewed beef	30 g	5.9 g Protein
		Meat samosa	45 g	8.5 g Protein
		Egg	50 g (1 egg)	6.4 g Protein
		Stewed omena (small fish)	25 g	6.8 g Protein
**Vegetables**	**80 g**	Stir-fried leafy vegetables	80 g	**weight of vegetables**
		Stir-fried cabbage	80 g	(Edible part)
		Pumpkin fruits	100g	
**Fruit**	**80-120 g**	Mango	100 g	**weight of fruits**
		Orange	100 g	(Edible part)
		Papaya	100 g	
		Pineapple	100 g	
		Sweet banana	100 g	
		100% fruits juice	200 g §	
		Avocado	100 g	
**Oil†**	**5 g**	**1 tsp of fat or oil**	**5 g**	**5 g Fat**
		Avocado	30 g	6 g Fat
		Dried groundnuts	10 g	14.5 g Fat
		French fried potato	50 g	6.8 g Fat
		Mandazi (doughnut)	50 g (1 piece)	6.5 g Fat
		Meat samosa	25 g (Half size)	5.0 g Fat
		Chapati	1/3 slice, 1 slice = 80 g	5.7 g Fat
**Sugar‡**	**5 g**	**1 tsp of Sugar**	**5 g Sugar**	**5 g Sugar**
		Milk tea	1 cup of milk tea	5 g Sugar
		Drinking chocolate	chocolate powder 10 g	
		Bottled soda / juice	1/7 bottle, 1 bottle = 300 ml	5.5 g Sugar

1) Defined by *National Guidelines for Healthy Diets and Physical activities (*2017), Ministry of Health Kenya.

2) Frequently consumed dishes in 24-hour dietary recall of present study.

†: Including oil-rich foods/dishes, ‡: Including sugar-sweetened foods/dishes, §: Half of the amount consumed is considered "fruit". Carb, carbohydrate; Ca, calcium; tsp, teaspoon.
